# Supplementary material for: Room-Temperature Optical Spin Polarization of an Electron Spin Qudit in a Vanadyl-Free Base Porphyrin Dimer
Source: J Am Chem Soc. 2024 Dec 16;147(1):331–41. doi: 10.1021/jacs.4c10632 (PMC11726572; doi:10.1021/jacs.4c10632)
Supplement: Supplementary file 1 — ja4c10632_si_001.pdf [file ja4c10632_si_001.pdf]

Supplementary Information for

Room-Temperature Optical Spin Polarization of an Electron Spin Qudit in a Vanadyl − Free Base Porphyrin Dimer

Alberto Privitera,[1,2*] Alessandro Chiesa,[3] Fabio Santanni,[4] Angelo Carella,[1,5] Davide Ranieri,[4] Andrea Caneschi,[2] Matthew D. Krzyaniak,[1] Ryan M. Young,[1] Michael R. Wasielewski,[1*] Stefano Carretta,[3*] and Roberta Sessoli[4*]

[1] *Department of Chemistry, Center for Molecular Quantum Transduction, and Paula M. Trienens Institute for Sustainability and Energy, Northwestern University, 60208-3113, Evanston, IL (USA)*

[2] *Department of Industrial Engineering, University of Florence & UdR INSTM Firenze, 50139, Firenze, Italy*

[3] *Department of Mathematical, Physical and Computer Sciences, University of Parma & UdR INSTM, 43124, Parma, Italy.*

[4] *Department of Chemistry "U. Schiff", University of Florence & UdR INSTM Firenze, 50019, Sesto Fiorentino, Italy*

[5] *Department of Chemical Sciences, University of Padova, 35134, Padua, Italy*

Table of Contents

1. Materials and methods **3**

2. Transient absorption (TA) spectroscopy **7**

3. Time-resolved Electron Paramagnetic Resonance (TREPR) **12**

4. TREPR in 5CB liquid crystal **15**

5. Simulation of TREPR assuming population of the doublet **16**

6. Continuous-Wave Electron Paramagnetic Resonance (CWEPR) **17**

7. Bibliography **18**

1. Materials and methods

Synthesis and characterization. The free-base monomer, H_2TrPP (**FP**), was purchased from *Porphychem sas* and used as such without further purification. Compounds [VO(TrPP)] (**VO**) and [$VOH_2(DPP)_2$] **(VO-FP)** were synthesized according to the procedure in refs.[1-2] The synthetic procedure for **VO-FP** is reported below for clarity. [VO(DPP)] (H_2DPP = 5,15-diphenylporphyrin) and H_2DPPBP = 5,15-diphenyl-10-(4,4,5,5-tetramethyl-1,3,2-dioxaboran-2-yl)-porphyrin precursors were synthesized as reported in ref.[1] Glassware was dried by heating at 120°C in vacuum. Anhydrous CH_2Cl_2 and DMF were purchased from ACROS Organics (AcroSeal) and used without further purification. All the manipulations involving moisture- and oxygen-free conditions were performed using standard Schlenk techniques and Ar as inert carrier. Mass spectrometry (MS) and nuclear magnetic resonance spectroscopy on proton (^{1}H-NMR) characterizations are reported here for completeness (Figures S1 – S5). ^{1}H-NMR analyses were performed on liquid solutions in $CDCl_3$ (Merck) with a Bruker Advance (400 MHz) instrument and Bruker AV 600 (600 MHz) instruments. Mass spectrometry analyses were carried out using the ThermoFisher LCQ Fleet Ion Trap LC/MS and the Bruker micrOTOF-Q™ III ESI-TOF Mass Spectrometry System equipped with the APCI source.

(5-[Oxo(10,20-diphenylporphyrinato-5-yl)vanadium(IV)]-10,20-diphenylporphyrin, V^{IV}O-free base dimer (VO-FP). [VO(DPP)] (10 mg, 15 μmol), H_2DPPBPin (5 mg, 15 μmol), and Cs_2CO_3 (13 mg, 40μmol) were added together to a flask, pumped in vacuum for 30 minutes, and put under Ar. The solids were then dissolved in a mixture of of dry DMF and toluene (5 mL + 10 mL). The solution was degassed by bubbling Ar for 45 min, and then, $Pd^0(PPh_3)_4$ (1.2 mg, 1 μmol) was added. The reaction mixture was heated to 80 °C and stirred for 18 h. Once returned to room temperature, the reaction was quenched by adding 10 mL of water. The mixture was extracted with CH_2Cl_2, washed with water, and the organic phase collected and dried on Na_2SO_4. The crude product was purified by flash chromatography on silica, using 1:1 hexane/CH_2Cl_2 as the eluent (flash SiO_2, R_f = 0.24). To remove residual [VO(DPP)], an additional column using toluene as eluent was performed (flash SiO_2, R_f = 0.67). After removal of the solvent in vacuum, the overall process yielded 65 mg of a purple microcrystalline powder (Yield: 83 %). ^{1}H NMR (400 MHz, CDCl3): δ 10.40 (s, 1H), 9.45 (br, 2H), 9.09 (br, 2H), 8.24 (br, 4H), 7.68-7.80 (m, 10H), −1.59 ppm (s, 2H). ESI-MS (m/z): 988.2843 ($M+H^+$, calcd), 988.2849 ($M+H^+$, found). IR $\tilde{\nu}_{max}/cm^{-1}$: 3302 (w, N-H, stretching), 3050 (w, C=C stretching), 3020 (w), 1597 (w, N=C stretching), 1317 (m), 1152 (w), 1065 (m), 997 (s, V=O S3 stretching), 967 (m), 957 (m), 856 (m), 792 (s), 722 (s), 697 (m), 655 (m). UV/Vis (CH_2Cl_2): λ_{max} (log(ε)) = 416 (5.3), 447 (5.3), 513 (4.7), 548 (4.7), 586 (4.3), 642 nm (3.4).

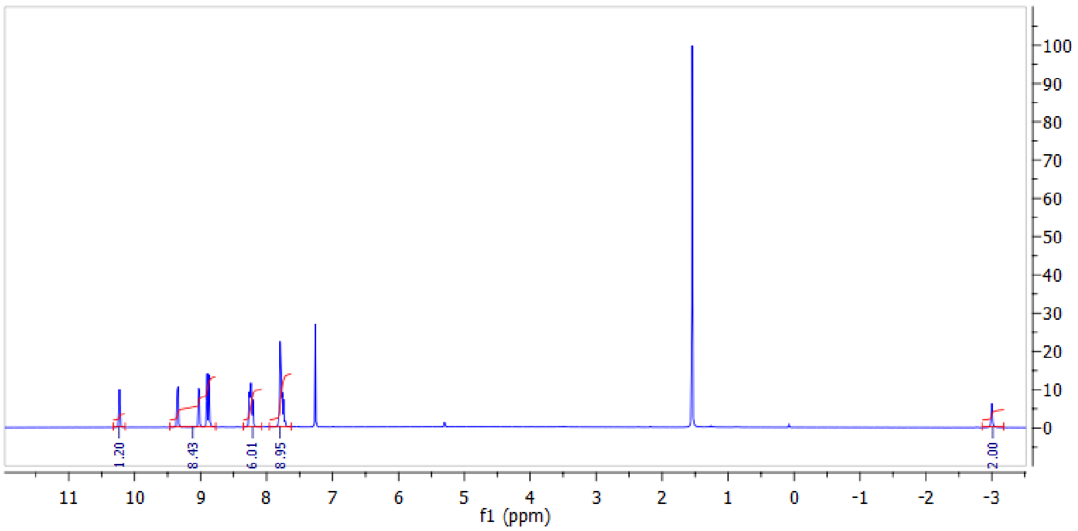

Figure S1. ^{1}H-NMR spectrum of **FP** in $CDCl_3$ in the range -3.5 – 12 ppm. Non-integrated peaks are ascribed to CH_2Cl_2 (5.30 ppm), H_2O (1. 55 ppm), and silicone grease (0.07 ppm) contaminations.

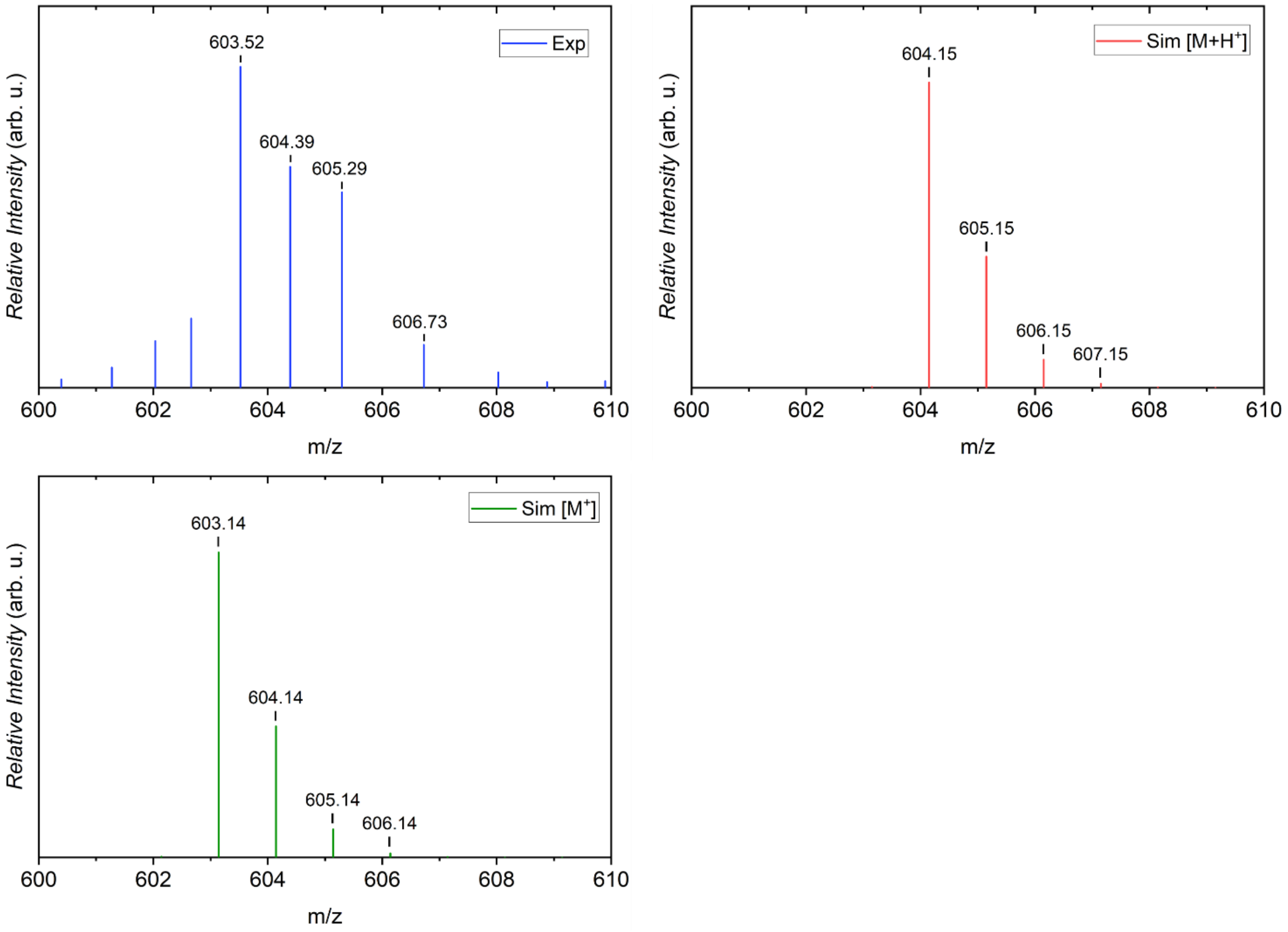

Figure S2. Experimental and simulated ESI-MS spectra of **VO**. Small discrepancies in **VO** isotopic pattern can be ascribed to different contributions to the overall spectrum from $[M]^+$ and $[M+H]^+$ species.

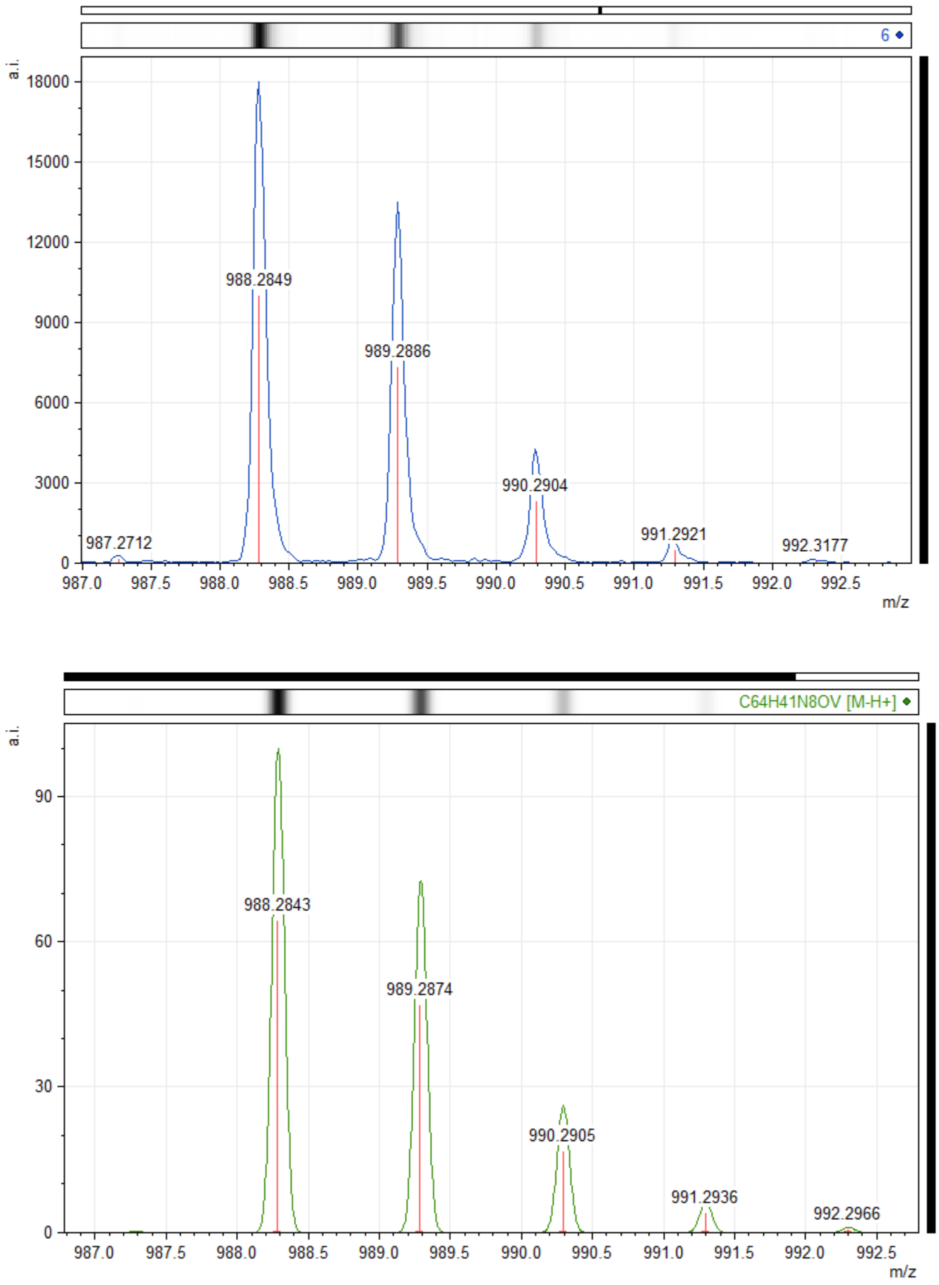

Figure S3: Experimental (top, blue line) and simulated (bottom, green line) APCI-MS signals of **VO-FP**.

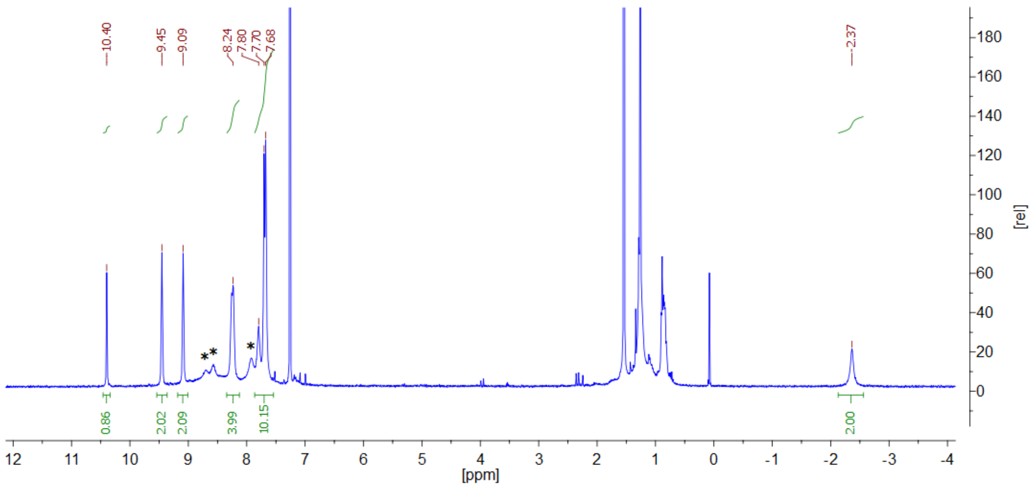

Figure S4: ^{1}H-NMR spectrum in $CDCl_3$ of **VO-FP**. The signals between 0 and 3 ppm correspond to minor solvent contamination.

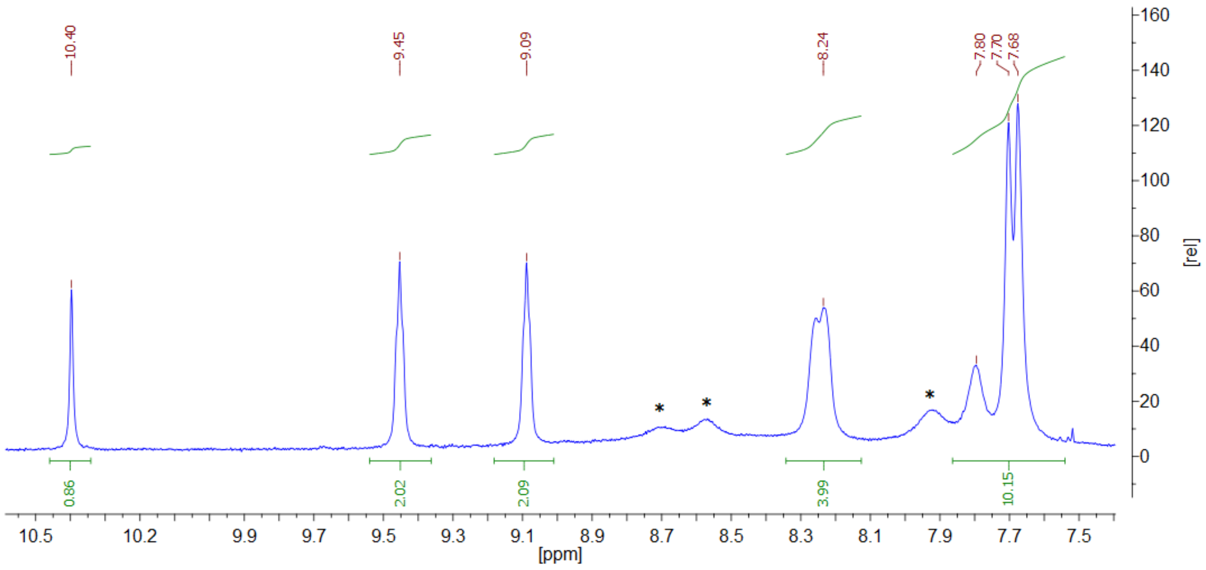

Figure S5: ^{1}H-NMR signals in $CDCl_3$ of compound **VO-FP** in the aromatic region. Only the signals ascribed to the free-base porphyrin unit were integrated. Broad ^{1}H signals of the vanadyl-porphyrin moiety in **VO-FP** are broadened by the presence of the paramagnetic center, and the few visible peaks are highlighted by an asterisk.

2. Transient absorption (TA) spectroscopy

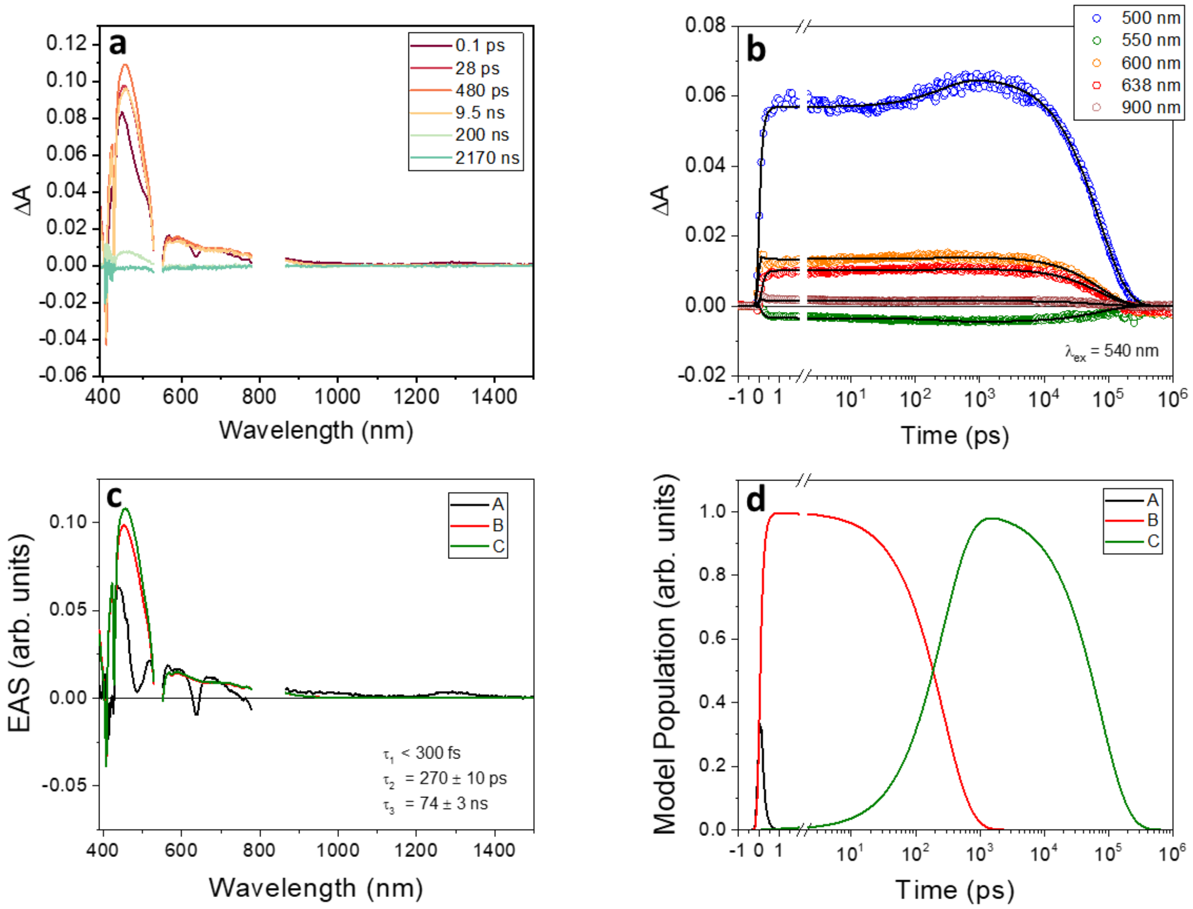

Figure S6. (a) Room-temperature fs/nsTA spectra of **VO** in toluene excited at 540 nm and recorded at selected delay times. (b) Selected wavelength kinetic fits, (c) evolution-associated spectra (EAS) and (d) population dynamics obtained by globally fitting the fs/nsTA data. The mechanism assumed to fit the data is A→B→C→ground state, where state A represents the singlet excited state of the porphyrin ligand. The EAS for states B and C look similar, differing only in amplitude and kinetics. This indicates that the model states B and C represent the same electronic state – in this case the porphyrin triplet – and that the transition represents a population loss associated with some molecular motion. Thus, we attributed B to the unrelaxed triplet state of the porphyrin ligand, and C to the fully relaxed triplet state of the porphyrin ligand.

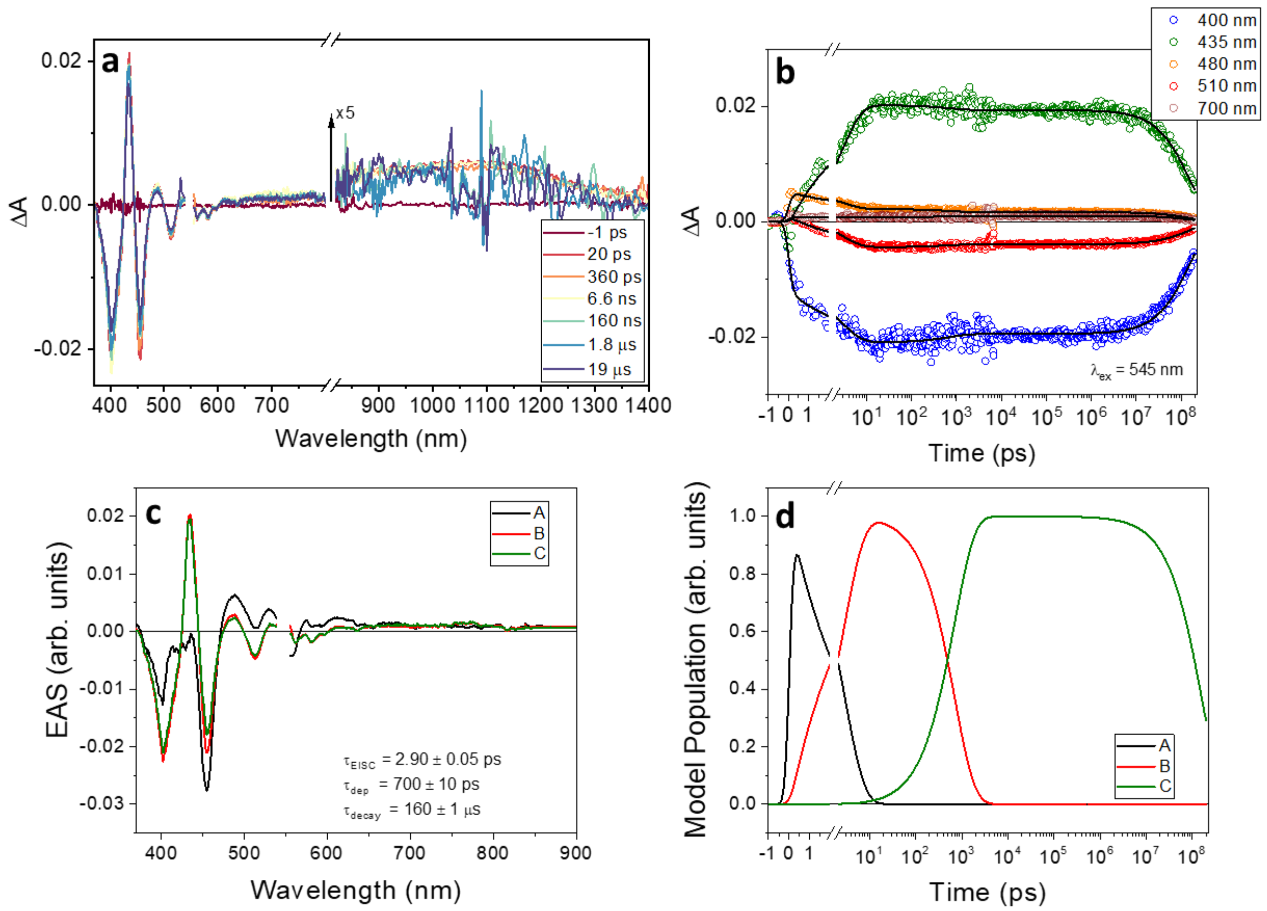

Figure S7. (a) Low-temperature (85 K) fs/nsTA spectra of **VO-FP** in butyronitrile excited at 545 nm and recorded at selected delay times. (b) Selected wavelength kinetic fits, (c) evolution-associated spectra (EAS) and (d) population dynamics obtained by globally fitting the fs/nsTA data. The mechanism assumed to fit the data is A→B→C→ground state, where state A represents the singlet excited state of FP, B is the unrelaxed triplet of FP, and C is the geometry-relaxed triplet state of FP.

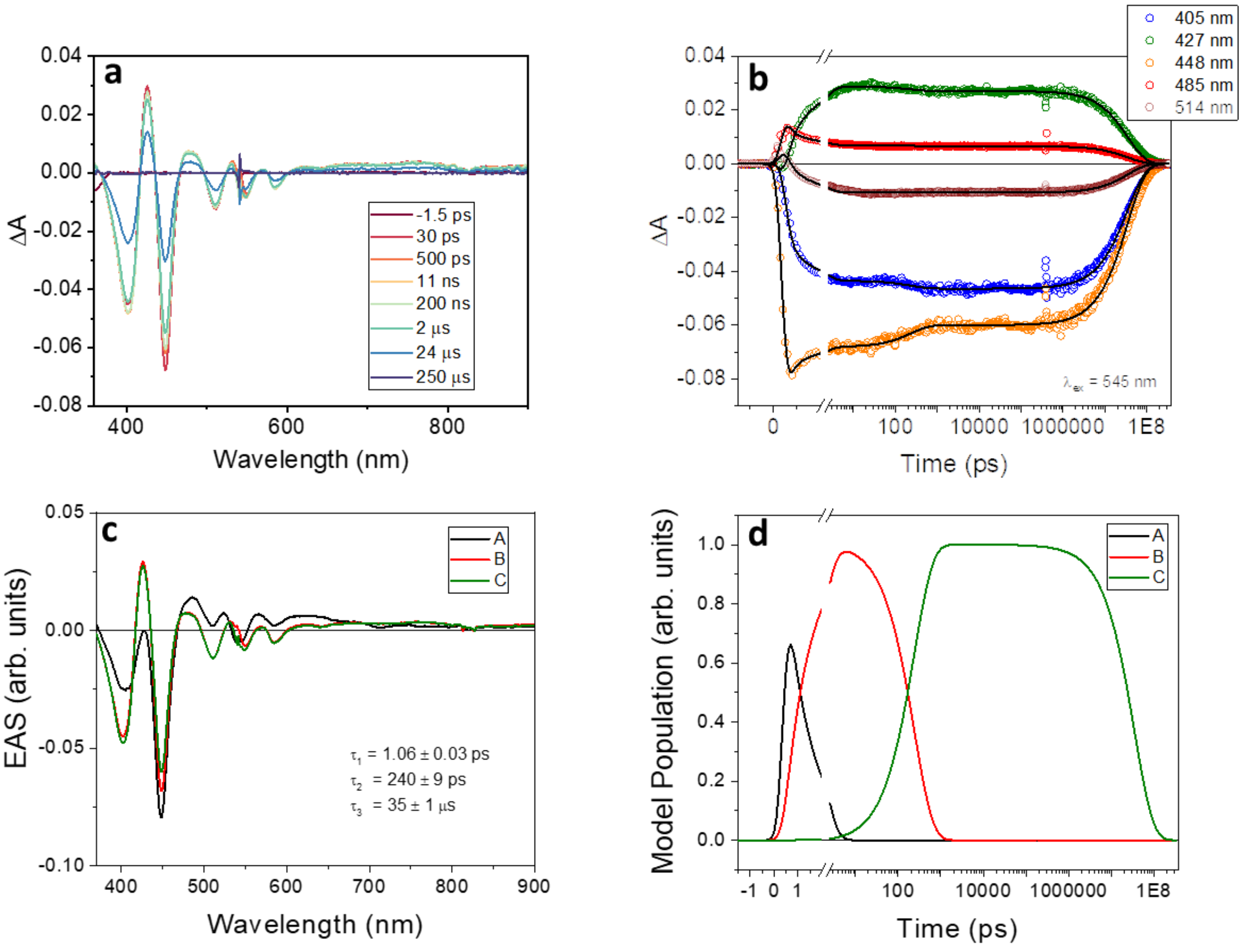

Figure S8. (a) Room-temperature fs/nsTA spectra of **VO-FP** in toluene excited at 545 nm and recorded at selected delay times. (b) Selected wavelength kinetic fits, (c) evolution-associated spectra (EAS) and (d) population dynamics obtained by globally fitting the fs/nsTA data. The mechanism assumed to fit the data is A→B→C→ground state, where state A represents the singlet excited state of FP, B is the unrelaxed triplet of FP, and C is the geometry-relaxed triplet state of FP. Importantly, no significant photophysical difference is observed between excitation at 640 nm and 545 nm. At 545 nm, light likely co-excites both the VO and FP units. In this scenario, various competitive mechanisms may occur, including vibrational relaxation of FP,[3] energy transfer from VO to FP,[4] and ISC in VO followed by energy transfer.[4] These processes likely take place within the time resolution of our TA setup. The only notable difference is a slowdown of the time constants when exciting at 640 nm compared to 545 nm, attributed to slightly faster dynamics at higher photon energies.

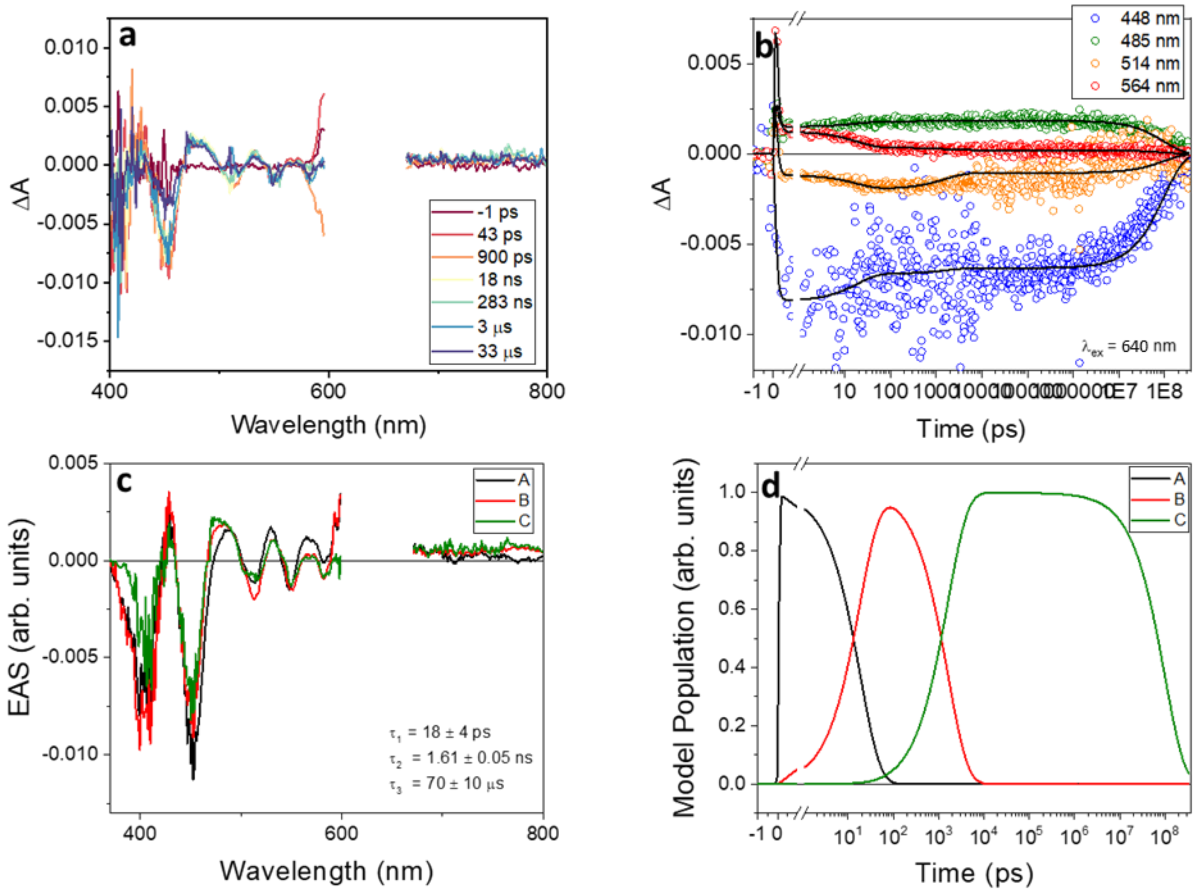

Figure S9. (a) Low-temperature (125 K) fs/nsTA spectra of **VO-FP** in toluene excited at 640 nm and recorded at selected delay times. (b) Selected wavelength kinetic fits, (c) evolution-associated spectra (EAS) and (d) population dynamics obtained by globally fitting the fs/nsTA data. The mechanism assumed to fit the data is A→B→C→ground state, where state A represents the singlet excited state of FP, B is the unrelaxed triplet of FP, and C is the geometry-relaxed triplet state of FP. Due to the high laser pump fluence (2 μJ/pulse) used, a relatively intense solvent response at short times is evident. Importantly, no significant photophysical difference is observed between excitation at 640 nm and 545 nm. The only notable difference is a slowdown of the time constants when exciting at 640 nm compared to 545 nm, attributed to slightly faster dynamics at higher photon energies.

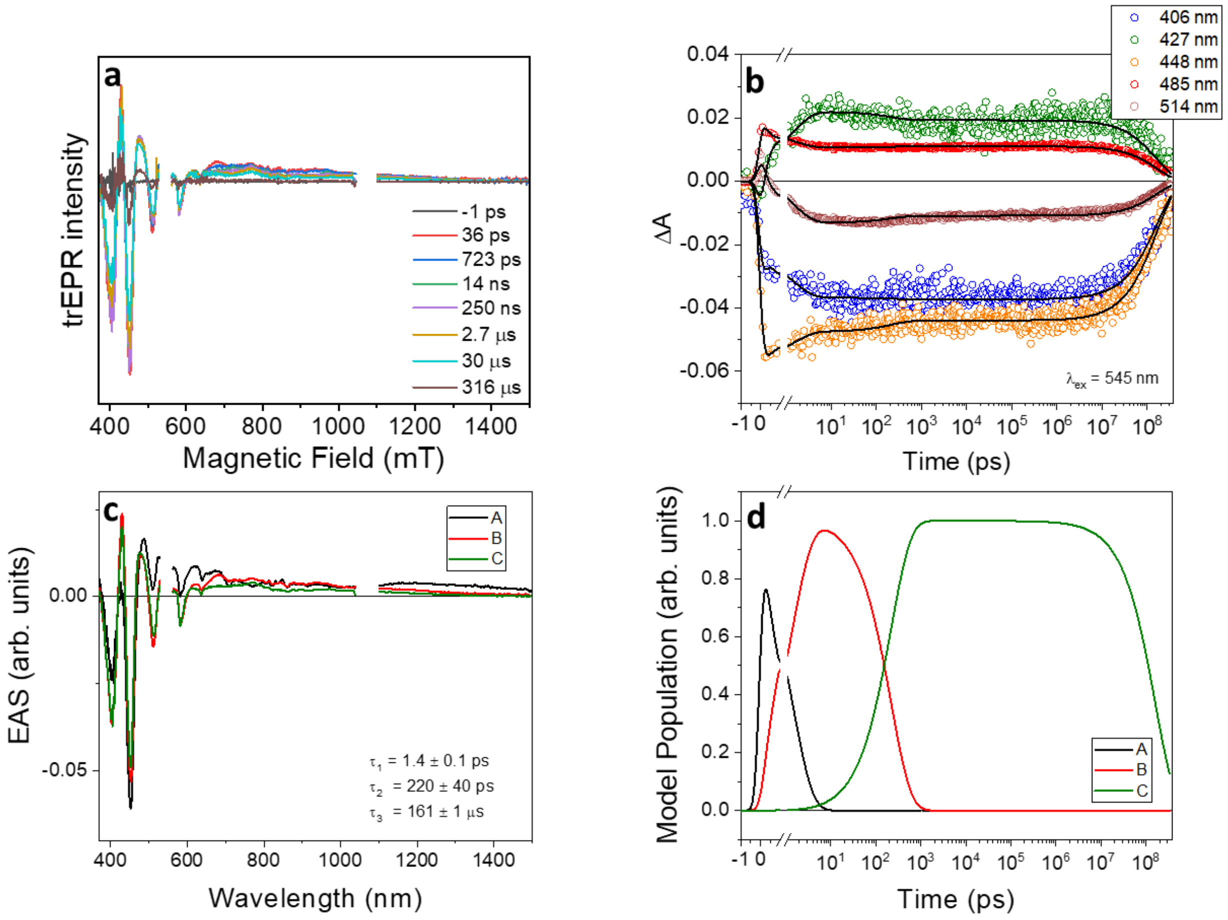

Figure S10. (a) Low-temperature (125 K) fs/nsTA spectra of **VO-FP** in toluene excited at 545 nm and recorded at selected delay times. (b) Selected wavelength kinetic fits, (c) evolution-associated spectra (EAS) and (d) population dynamics obtained by globally fitting the fs/nsTA data. The mechanism assumed to fit the data is A→B→C→ground state, where state A represents the singlet excited state of FP, B is the unrelaxed triplet of FP, and C is the geometry-relaxed triplet state of FP.

3. Time-resolved Electron Paramagnetic Resonance (TREPR)

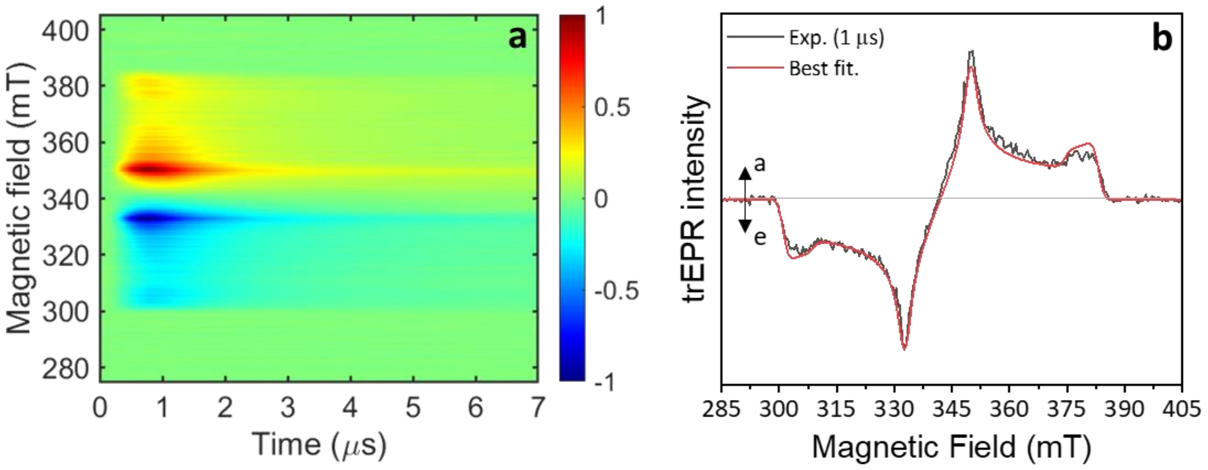

Figure S11. (left) Normalised 2D experimental TREPR contour plots of **FP** in toluene acquired at 85 K after a 550 nm laser pulse (7 ns, 2 mJ). Colour legend: red = enhanced absorption, blue = emission, green = baseline. (right) Normalised 1D experimental TREPR spectrum (black line) and best-fit spectral simulation (red line) of H_2TrPP taken at 1 μs after the laser pulse. Arrows legend: a = enhanced absorption, e = emission.

Table 1. Best-fit simulations values obtained from the TREPR spectral simulation in Figure S11.

g	2.002
[D, E] (MHz)	[1153, -224] ± 5
[p_x p_y p_z]	[0.29 0.71 0] ± 0.02
LW (mT)	2.5 ± 0.2

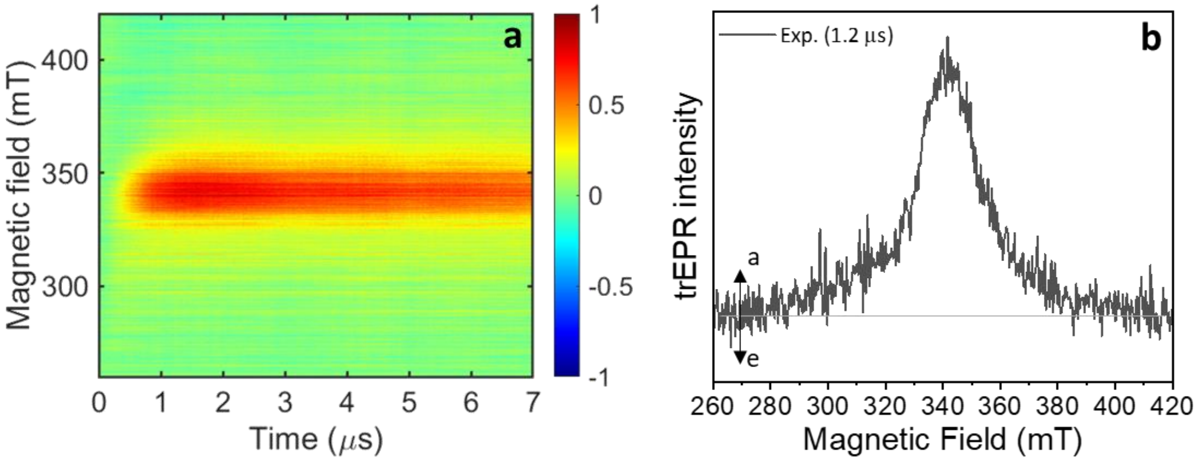

Figure S12. (a) Normalized 2D experimental TREPR contour plots of **VO** in toluene acquired at 85 K after a 550 nm laser pulse (7 ns, 2 mJ). Color legend: red = enhanced absorption, blue = emission, green = baseline. (b) Normalized 1D experimental TREPR spectrum of VOTrPP taken at 1.2 μs after the laser pulse. Arrows legend: a = enhanced absorption, e = emission.

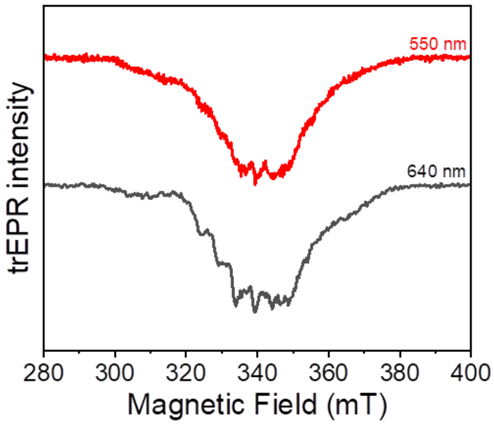

Figure S13. Comparison between normalized 1D experimental TREPR spectra of **VO-FP** taken at 1.2 μs after 550 nm (red line) and 640 nm (black line) laser pulses. The comparison reveals negligible difference between the two spectra, with the exception of a minor line broadening observed in the 550 nm spectrum.

4. TREPR in 5CB liquid crystal

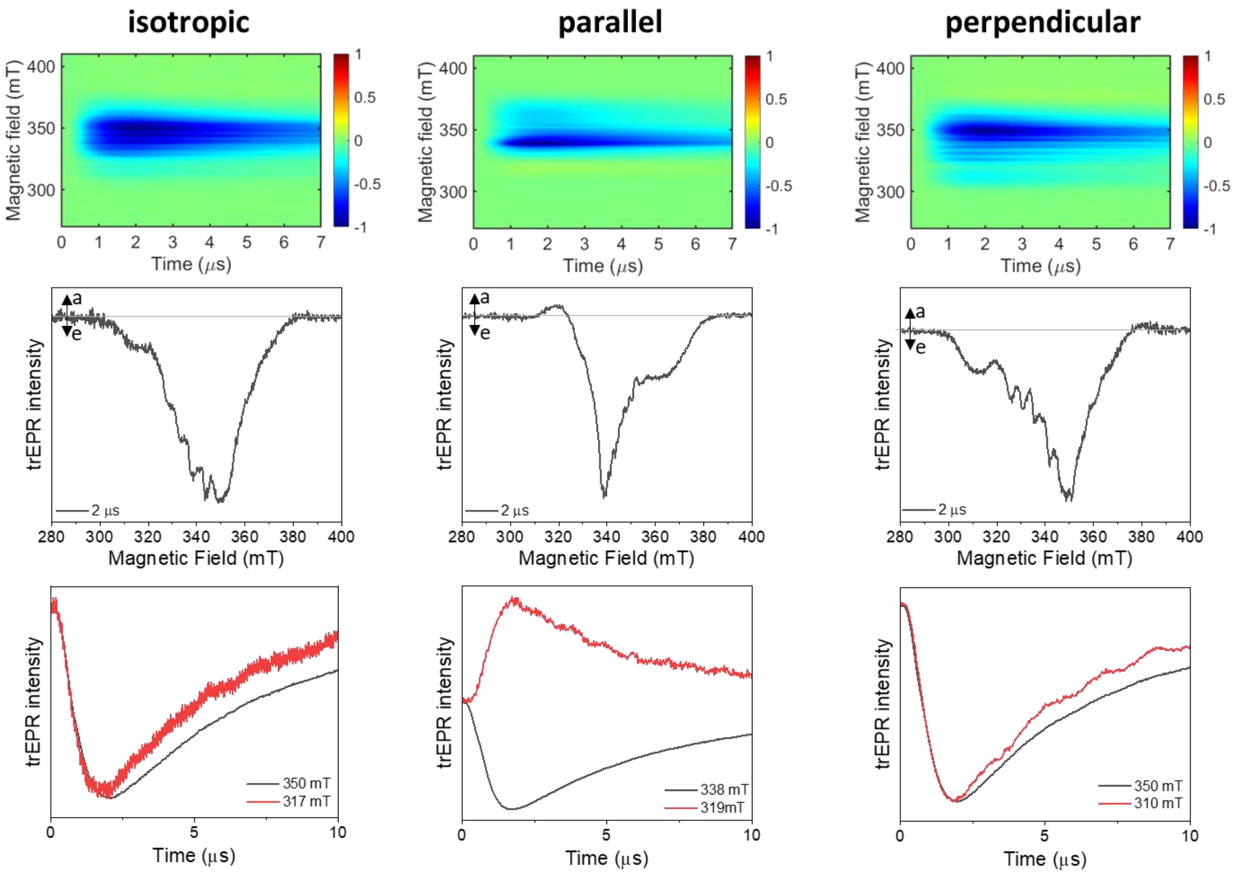

Figure S14. (top) Normalized 2D experimental TREPR contour plots of **VO-FP** oriented in the nematic liquid crystal 5CB at 85 K acquired after a 640-nm laser pulse (7 ns, 2 mJ). The long axis of each molecule is aligned at 0° (parallel) and 90° (perpendicular) relative to the applied magnetic field direction. Color legend: red = enhanced absorption, blue = emission, green = baseline. (center) Normalized 1D experimental TREPR spectra taken at 2 μs after the laser pulse. Arrows legend: a = enhanced absorption, e = emission. (bottom) TREPR transients taken at two relevant magnetic field points.

5. Simulation of TREPR assuming population of the doublet

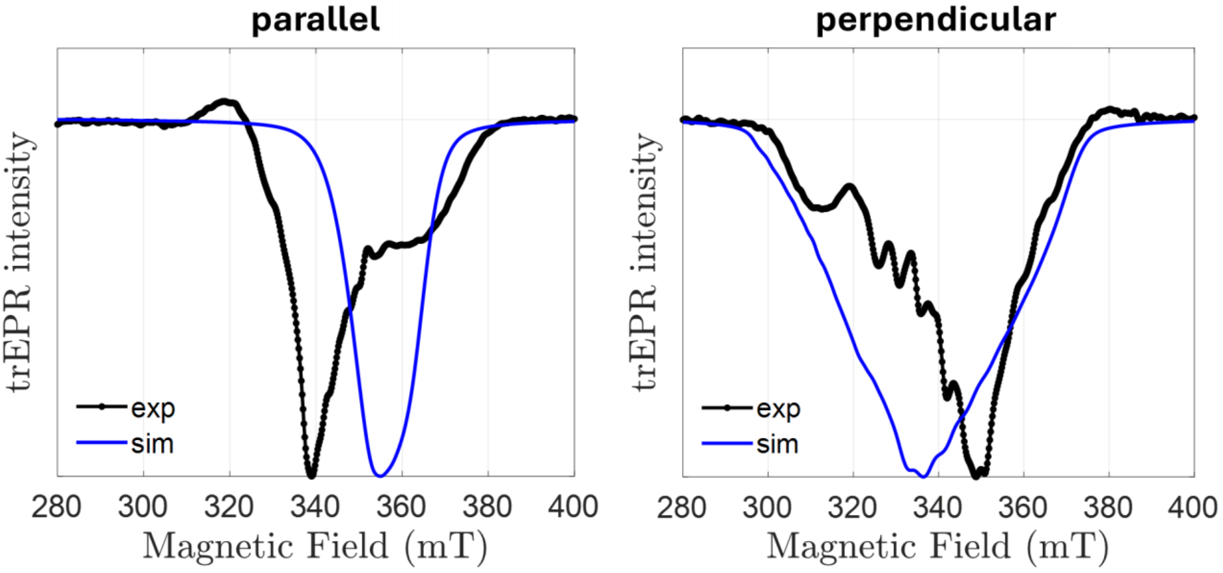

Figure S15. Simulation of the TREPR spectrum for parallel (left) and perpendicular (right) orientations of the molecule relative to the applied field, assuming the initial population of the trip-doublet state and using the known spin Hamiltonian parameters from the main text. No free parameters were used for electron spin polarization, and nuclear spin polarization was omitted for simplicity (i.e., $\rho_N = \frac{I}{8}$). The experimental spectrum in the parallel orientation is notably narrower than the simulated one, and the main minimum is shifted by 10-15 mT in opposite directions for the two orientations. Adjusting the populations of nuclear spin sublevels can alter the lineshape but does not affect these conclusions regarding the spectrum center and width. These simulations rule out the possibility of a significant contribution from a long-lived trip-doublet state in the EPR spectrum.

6. Continuous-Wave Electron Paramagnetic Resonance (CWEPR)

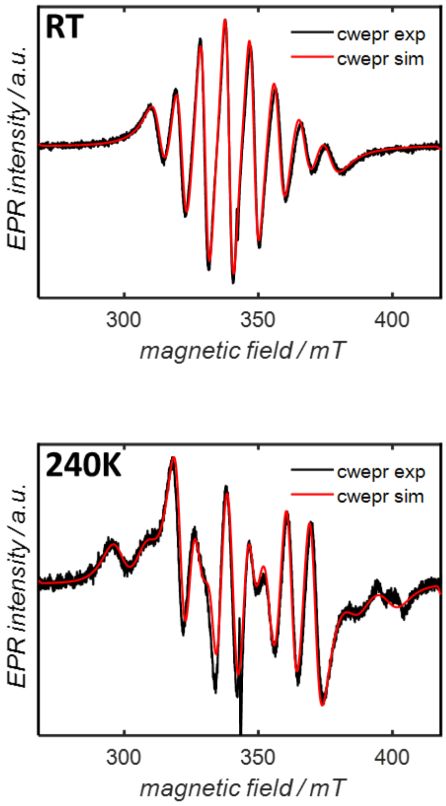

Chili – slow-motional regime
Sys.S = 1/2;
Sys.g = [1.985 1.985 1.964];
Sys.Nucs = '51V';
Sys.A =[162 162 475];
Sys.lw = 1.5;
Sys.tcorr = 2.1e-10; % = 0.21 ns

Chili – slow-motional regime
Sys.S = 1/2;
Sys.g = [1.985 1.985 1.964];
Sys.Nucs = '51V';
Sys.A =[162 162 475];
Sys.lw = 1.3;
Sys.tcorr = 8e-10; % = 0.8 ns

Figure S16. (left) CWEPR spectra of **VO-FP** (black line) in toluene acquired at room temperature and 240 K. The spectral simulations (red line) were obtained using the routine chili of the Matlab toolbox Easyspin.[5] The simulation parameters used for the simulation are reported on the right.

7. Bibliography

1. Ranieri, D.; Privitera, A.; Santanni, F.; Urbanska, K.; Strachan, G. J.; Twamley, B.; Salvadori, E.; Liao, Y.-K.; Chiesa, M.; Senge, M. O.; Totti, F.; Sorace, L.; Sessoli, R., A Heterometallic Porphyrin Dimer as a Potential Quantum Gate: Magneto-Structural Correlations and Spin Coherence Properties. *Angew. Chem. Int. Ed.* **2023,** *62*, e202312936.

2. Ranieri, D.; Santanni, F.; Privitera, A.; Albino, A.; Salvadori, E.; Chiesa, M.; Totti, F.; Sorace, L.; Sessoli, R., An exchange coupled meso–meso linked vanadyl porphyrin dimer for quantum information processing. *Chem. Sci.* **2023,** *14*, 61-69.

3. Baskin, J. S.; Yu, H.-Z.; Zewail, A. H., Ultrafast Dynamics of Porphyrins in the Condensed Phase: I. Free Base Tetraphenylporphyrin. *J. Phys. Chem. A* **2002,** *106*, 9837-9844.

4. Asano-Someda, M.; van der Est, A.; Krüger, U.; Stehlik, D.; Kaizu, Y.; Levanon, H., Intramolecular Energy Transfer in a Covalently Linked Copper(II) Porphyrin−Free Base Porphyrin Dimer: Novel Spin Polarization in the Energy Acceptor. *J. Phys. Chem. A* **1999,** *103*, 6704-6714.

5. Stoll, S.; Schweiger, A., EasySpin, a comprehensive software package for spectral simulation and analysis in EPR. *J. Magn. Reson.* **2006,** *178*, 42-55.
